# Discovering the stacking landscape of a pyridine-pyridine system

**DOI:** 10.1007/s00894-017-3496-4

**Published:** 2017-11-09

**Authors:** Tomasz Sierański

**Affiliations:** Institute of General and Ecological Chemistry, Lodz University of Technology, Zeromskiego 116, 90-924 Lodz, Poland

**Keywords:** Stacking interactions, Pyridine, Density-functional theory, Energy decomposition analysis, Natural bond orbital analysis

## Abstract

Extremely extensive calculations of potential energy surfaces for the parallel-displaced configuration of pyridine dimer systems have been carried out using a dispersion-corrected density functional. Instead of focusing on stationary geometries these calculations provide much deeper insight into the “landscape” of the interaction energies of the particular systems—one can learn how the pyridine dimer stability changes along with various geometrical parameters. Other calculations such as natural bond orbital and energy decomposition have also been applied. The interplay of two significant factors, electrostatic forces and electron correlation effects, have been evaluated. The role of π···π interactions in the stacked pyridine systems has also been confirmed, and surprisingly, this happened to be true even for the geometries where the formation of C-H···π interactions might be proposed instead. The combination of many different methods has revealed the complexity of the stacking interactions. Apart from providing a “literal new look” into pyridine interaction patterns another picture has emerged. A stacking interaction in a pyridine dimer system is perceived as a combination of many different sources of the interaction energy, including orbital ones, and this is true for many different geometries.

**Electronic supplementary material:**

The online version of this article (10.1007/s00894-017-3496-4) contains supplementary material, which is available to authorized users.

## Introduction

Noncovalent interactions (NCI hereafter) in aromatic ring systems are significant in many areas of science. They play a key role in the structures of biomolecules, molecular recognition, material science, and nanoengineering [1–8]. This is especially true for the N-heterocyclic ring system. The presence of a nitrogen atom in their structures enriches their ring framework π-cloud with electrons and enables their participation in different interaction patterns (e.g., C-H···N hydrogen bonds) [9–13]. Thus, N-heterocycles may be considered as useful recognition elements in many biological systems. They are also used as building blocks for anchoring substituents in defined position and become a key component in most known drug molecules [9, 14–16]. Though the study of stacking interactions has mostly been focused on a benzene molecule and its simple derivatives [17–23], the number of studies of such interactions in the case of heterocyclic ring systems have been quickly increasing [9, 11, 24–29].

The most studied systems containing N-heterocycles are dimers containing pyridine molecules [10, 26, 27, 30–37]. The studies, in general, are focused on determination of system energy as a function of selected geometrical parameters but other approaches such as full geometry optimizations, starting from various unsymmetrical initial dimer arrangements, have also been applied [38]. In addition, quantum chemistry calculations have recently been combined with the statistical analysis based on data from the Cambridge Structural Database (CSD) [10, 26, 39–42]. This combination study appeared to be very useful and helped to learn a lot of the stacking interaction involving pyridine dimer systems (e.g., the existence of binding interaction for ring centroid distances larger than 6 Å) [10, 40]. Though some significant progress has definitely been made, most studies only focus on analyzing stationary geometries and say nothing of a whole range of other configurations. The role of the molecular geometrical dependencies on the intermolecular interaction of the stacked systems is not fully understood and the exact binding geometrical boundaries (the boundaries between positive and negative interaction energies) of the stacked pyridine systems are not known since the accurate potential energy maps of these systems, based on several geometrical parameters, are not available. Some attempts have been made before, but the obtained PES map for a pyridine dimer was restricted only to two geometrical parameters with a relatively large distance step (the distance between pyridine monomers was changed with 1 Å increments) [10]. Accurate PES maps would be very useful in providing information about the key importance of the complete understanding of the stacking interaction phenomenon, literally giving a new way to look into the stacked interactions patterns of pyridine systems. Instead of analyzing simple potential energy curves one could see a wider picture of what really occurs and learn how the pyridine dimer stability changes along with various geometrical parameters. A whole area of pyridine dimers possessing similar energy could be observed and one could know how one interaction, as different geometrical parameters change, transforms into another. Going beyond the analysis of the stationary geometries, multidimensional, accurate potential energy maps could find a very broad range of applications in chemistry. They would appear to be a guide for qualitative predictions of binding interactions and could help to learn more of the influence of the position of nitrogen atoms and C-H bonds (e.g., the rotation of one monomer around the other) on the dimer system energy as well as determine the geometrical regions in which the interaction energy stays the same or changes negligibly, helping to harness the full potential of NCI (e.g., in predicting and understating the molecular packing patterns as well as in drug design strategy). This is true not only for pyridine based systems as the obtained knowledge may be transferred to other N-heterocycles. Moreover, when those multidimensional data are analyzed with the results of other methods, such as energy decomposition analysis methods and/or natural bond orbital analysis, they may disclose important aspects associated with the physics of NCI (e.g., the actual importance of π orbitals in intermolecular interactions in stacked systems).

Taking all the above into account, as the main focus of this study, the extremely extensive analysis of potential energy surfaces (PES) of model pyridine-pyridine dimers has been selected. The interaction energies of approximately 25,000 systems have been calculated. Because of the use of a pyridine ring as a versatile core in the pharmaceutical field [43], the presented study may also be relevant in electronic properties of pyridine-unit-based drugs.

The accurate calculations of NCI interaction energies require an appropriate description of the dispersion forces. Hence, the use of advanced wave function theory (WFT) methods is needed. Unfortunately, they are very expensive and cannot be applied for routine calculations [44]. An alternative to those WFT methods are density functional theory (DFT) methods whose cost is significantly lower. Recently, a number of new density functionals enabling the energy calculations of non-covalently bonded systems have been developed. One of the simplest ways of accounting for dispersion forces is to add a dispersion correction term. This method is robust, very fast, and it is easily programmable. Some of dispersion-corrected density functionals, with D3-correction [45, 46], give results that in many cases are close to the advanced WFT methods for dispersion-dominated NCI [45–47]. Apart from adding a dispersion correction term, it is also worth mentioning the hybrid meta exchange-correlation functionals developed by the Truhlar Group, among which one of the most popular is M06-2X as it performs well in the general chemistry of main group elements and enables a great improvement in calculations of NCI interaction energies [48, 49].

## Methods

**Fig. 1:**
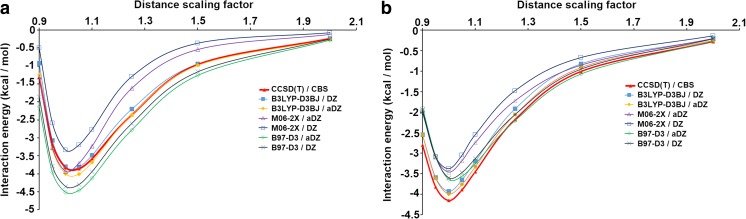
Interaction energy curves created on the basis of data from Sx66 data set [52, 53] for both stacked (**a**) and hydrogen bonded (**b**) pyridine dimer systems. Distance scaling factors are the factors used for scaling the closest intermolecular distance starting from the equilibrium geometries [52, 53]. The curves were created using splicing interpolation. CBS stands for complete basis set extrapolation

In order to identify an appropriate density functional for the PES study, the selected density functionals, based on the literature data [9, 48–51] were tested against the S66x8 data set [52] for both stacked and hydrogen bonded pyridine dimer systems (Fig. 1). All the necessary geometries were taken from the Benchmark Energy and Geometry Data Base [53]. In the selected functionals both aug-cc-pVDZ and cc-pVDZ (respectively aDZ and DZ hereafter) basis sets were used. Since the energies of a high number of systems were to be calculated, taking into account higher basis sets would make the calculations extremely costly and time-consuming. B97-D3, B3LYP-D3BJ, and M06-2X were selected as the tested DFT functionals. B97-D3 and B3LYP-D3BJ are a dispersion-corrected DFT with D3 version of Grimme’s dispersion with Becke–Johnson damping [46]. They have been shown to describe NCI in many systems with aromatic rings [9, 46, 51]. M06-2X was selected due to its popularity and its good performance in calculation of NCI between aromatic molecules [48, 49]. Among these functionals, B3LYP-D3BJ appeared to be the best. It gave excellent results for both hydrogen bonded and stacked pyridine dimers (Fig. 1). For this functional, the influence of diffuse functions (aDZ vs DZ) on the pyridine system energy was negligible with the mean deviations from the reference CCSD(T) (coupled cluster with single, double, and perturbative triple excitation contributions) [44] equal to 0.12 and 0.16 kcal mol^-1^, respectively for aDZ and DZ (Fig. 1). The remaining functionals performed worse with the mean deviations values equal to 0.40, 0.65, 0.43, and 0.38 kcal mol^-1^, respectively for M06-2X/aDZ, M06-2X/DZ, B97-D3/aDZ, and B97-D3/DZ. It is also seen that in the case of M06-2X, the lack of diffuse functions significantly worsens the NCI energy calculations (Fig. 1). Taking this into account, the general performance and the calculation time, for all further calculations B3LYP-D3BJ with DZ was selected (the use of DZ instead of aDZ basis set significantly speeds up the computation) .

**Fig. 2:**
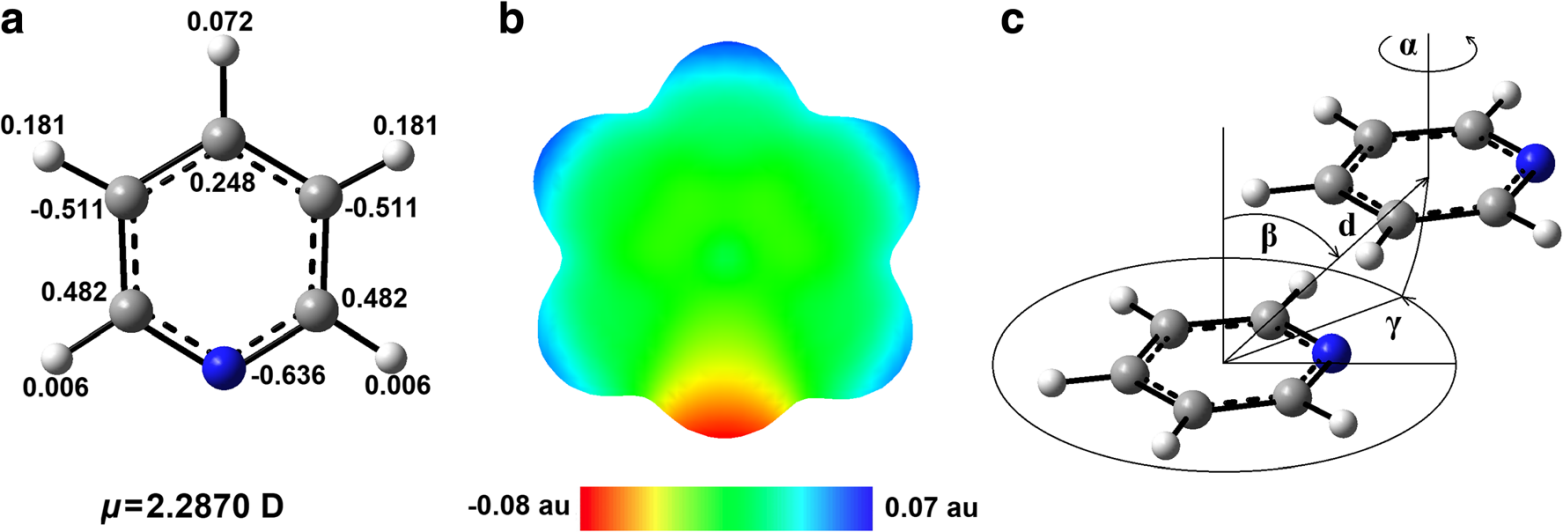
Atom HLY charge values (**a**), EPS map (**b**) calculated for a pyridine molecule and the geometrical model of a pyridine dimer (**c**) used in presented calculations (*d*, the aromatic ring center distance; *α*, the twist angle between the monomers; *β*, the angle between the line connecting the aromatic ring centers and the normal line to the aromatic ring in which the connecting center line starts). For all parameters equal to zero, the atom positions of one monomer are the same as the corresponding atom positions of the second one. HLY charge values (**a**) are given in *e* (elementary electric charge), the EPS map (**b**) is superimposed on the isodensity surface with 0.005 au

Model pyridine-pyridine systems were constructed as shown in Fig. 2. In each of them the pyridine rings were parallel to each other. PES scans were done as a function of four geometrical parameters: the aromatic ring center distance (*d*), the twist angle (*α*) between the pyridine rings, the angle between the line connecting the aromatic ring centers and the normal line to the aromatic ring in which the connecting center line starts (*β*) and the angle determining the rotation of one pyridine monomer around the other (*γ*). All calculations were performed using Gaussian09 rev. D.01 [54]. The starting monomer was a pyridine molecule with geometry optimized at MP2/aDZ level, where MP2 stands for second-order Møller-Plesset perturbation theory [55]. The dimer energies were corrected for basis set superposition error by the use of the counterpoise method [56]. Interaction energies were calculated by subtracting the energy of the two monomers:

$$ {\mathrm{E}}_{\mathrm{Interaction}}={\mathrm{E}}_{\mathrm{Dimer}}\hbox{--} {\mathrm{E}}_{\mathrm{MonomerA}}\hbox{-} {\mathrm{E}}_{\mathrm{MonomerB}} $$

The pyridine-pyridine system energies were calculated as a function of the selected geometrical parameters. The parameter *d* was varied from 3 to 7 Å with 0.1 Å increments, *α* was varied from 0 to 330° with 30° increments, *β* was varied from 0 to 90° with 10° increments and *γ* was varied from 0 to 180° with 45° increments. Because of the dimer system symmetry, taking into account higher *γ* values was not necessary. For *γ* = 0° the lateral shifting (associated with the increase in *d* values at the given *β*) of one ring along the plane of the other was in accordance with the direction of the vector created between the other ring centroid and the nitrogen atom of this ring (the vector beginning was the position of the ring centroid).

**Fig. 3:**
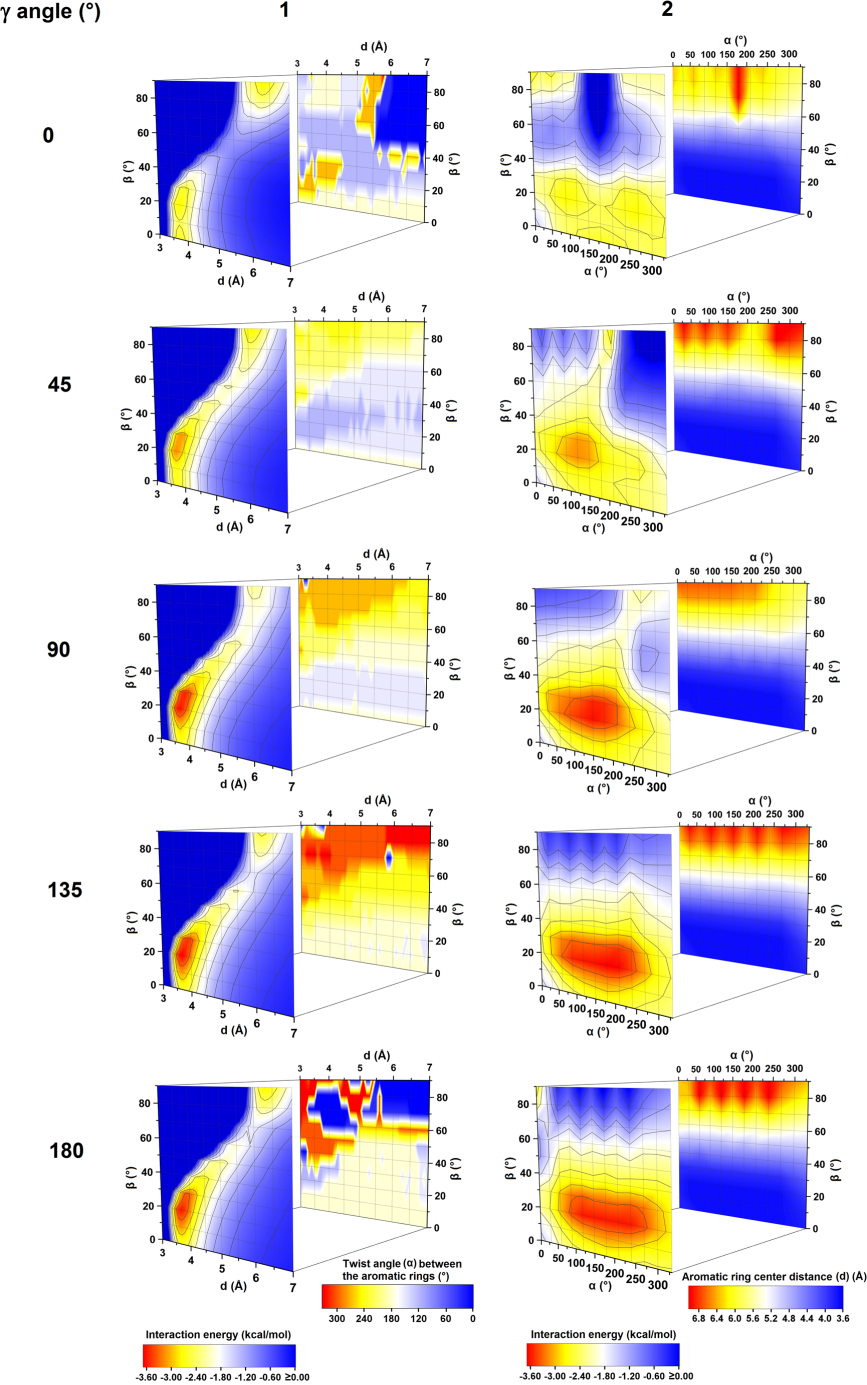
PES maps of the studied pyridine dimer systems created on the basis of energy minima found for the systems with the given *β* and *d* parameter values (1) as well as for the systems with the given *α* and *β* values (2). These PES maps are shown together with corresponding twist angles between the aromatic rings (1) and the corresponding aromatic ring centers distances (2)

**Fig. 4:**
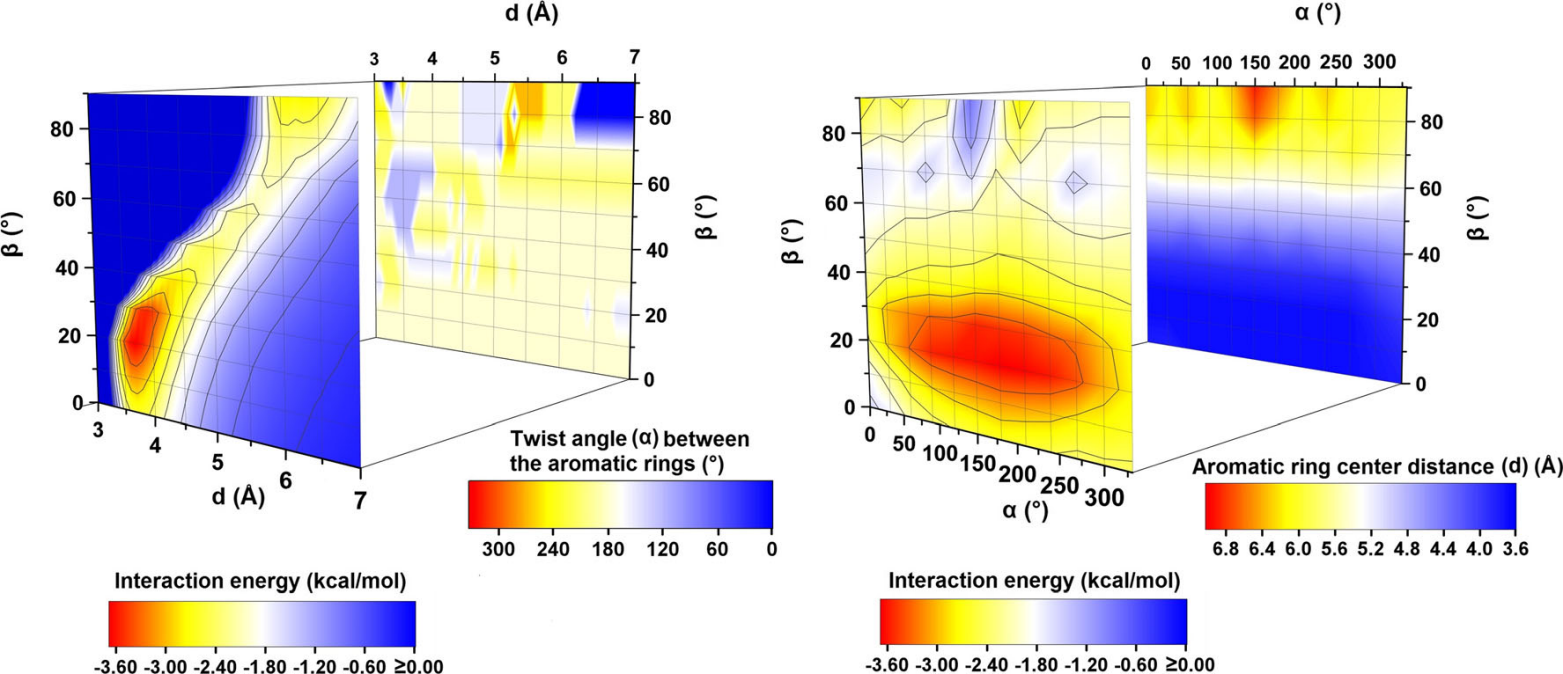
PES maps of the studied pyridine dimer systems along with the maps depicting the corresponding *α* (°) and *d* (Å) values. The PES maps were created on the basis of energy minima (kcal mol^-1^) found for the systems with the given *β* and *d* values (on the left) as well as for the systems with the given *α* and *β* values (on the right)

**Fig. 5:**
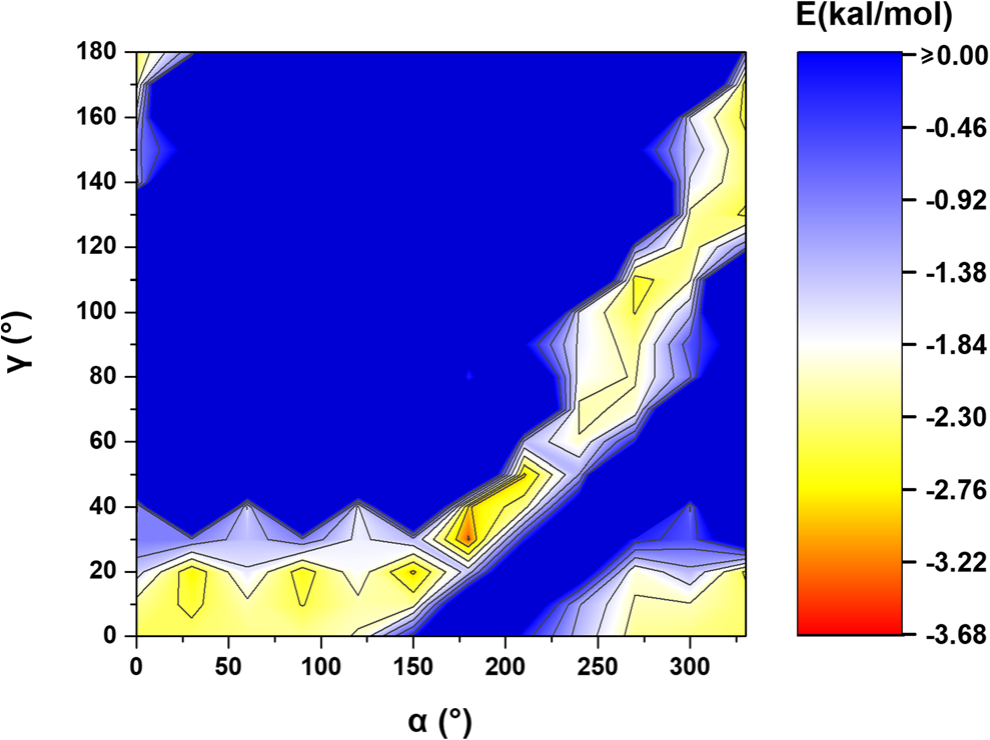
PES maps of the studied pyridine dimer systems created on the basis of energy minima found for the systems with the given *α* and *γ* parameters values and for *β* = 90° and *d* = 6.1 Å

On the basis of the obtained data (single points calculations for each system) the contour plots representing energy as a function of *α* and *β* and as a function of *d* and *β* (Figs. 3 and 4) were created using OriginPro 2016 [57]. The model pyridine dimers, whose geometries correspond to the found global and local energy minima on the created PES maps, were optimized (at B3LYP-D3BJ/DZ) to get more accurate system configurations. For *β* = 90° pyridine molecules, being coplanar, can create two C-H···N hydrogen bonds. For this configuration, the interaction energy between pyridine molecules also appeared to strongly depends on γ angle and a relatively large difference in energy between the non-optimized and optimized geometry was observed. Hence, an additional PES scan was made with *β* and *d* taken from the non-optimized geometry; *α* was varied from 0 to 330° with 30° increments and *γ* was varied from 0 to 180° with 10° (Fig. 5).

Some intermolecular interactions in the investigated pyridine-pyridine systems (dimers whose geometries correspond to the found global and local energy minima as well as those with geometries corresponding to PES map regions in which the interaction energy stays the same or changes negligibly) were analyzed by means of localized molecular orbital energy decomposition analysis (LMO-EDA) [58]. LMO-EDA can be considered as an extension and modification of the methods developed by Kitaura and Morokuma, Ziegler and Rauk, and Hayes and Stone [59–61]. This relatively new and robust method can be used with many quantum mechanical approaches (e.g., DFT and CC). Since its implementation in GAMESS software package [62, 63], LMO-EDA has been successfully used to study the intermolecular interactions (e.g., hydrogen bonds, π···π, and C-H···π contacts) in a number of systems such as pyrrole-pyrrole dimers [9], indole–cation–anion complexes [64], aromatic units of amino acids with guanidinium cation [65], hydrogen-bonded complexes of serotonin [66], radical conjugated systems [67], and C-H/π complexes in water [68]. The interaction energy in LMO-EDA is decomposed to electrostatic (E^ele^), exchange (E^ex^), repulsion (E^rep^), and polarization (E^pol^) terms and they are calculated on the basis of single-determinant Hartree–Fock (HF) wavefunctions. The correlation term (E^corr^), roughly dispersion, is derived from a supermolecule approach by the use of a correlation method (such as MP2 or CC) and it equals the difference between the correlation method energy and HF energy [58]. In this work, the contribution of correlation energy was estimated from B3LYP-D3BJ/DZ calculations and the E^corr^ was calculated as a difference between B3LYP-D3BJ/DZ energy and HF/DZ energy. The LMO-EDA calculations were made using GAMESS [62, 63] software employing the HF/DZ method. In the LMO-EDA calculation, as implemented in GAMESS, the counterpoise correction for basis set superposition error is used.

In order to find possible orbital interactions between the pyridine monomers, the electronic properties of the model pyridine dimers, whose geometries correspond to the found global and local energy minima as well as those with geometries corresponding to PES map regions in which the interaction energy stays the same or changes negligibly, were analyzed in terms of natural bond orbital (NBO) analysis with the use of hybrid meta exchange-correlation functional, M06-2X, employing aug-cc-pVTZ basis set. Those pyridine dimer geometries were also analyzed (at M06-2X/aug-cc-pVTZ level of theory) in terms of the Hu, Lu, and Yang (HLY) charge-fitting method [69]. This method appeared to give better results than the commonly accepted CHelpG scheme [69, 70]. M06-2X density functional was selected due to its excellent performance during application in the general chemistry of main-group elements [48, 49].

## Results and discussion

The performed calculations have enabled creation high quality PES maps that enable one to see how the pyridine dimer stability changes along with various geometrical parameters. On the basis of these maps the optimal configurations of the pyridine dimer may also be identified (Fig. 3). It is also observed that the binding boundaries on the PES maps are relatively broad and they are seen for different geometrical parameters.

The minimum *d* set as 3 Å is too small to provide binding interaction energies (negative ones) in pyridine dimers. Generally, with the increase in *d* the interaction energy between the pyridine monomers decreases which encourages the pyridine molecules to bind to each other (Figs. 3 and 4). However, an optimal system configuration cannot be achieved by the monomers separation alone and other geometrical parameters need to be changed as well. Hence, a sandwich configuration is usually not associated with any energy minimum since it involves too much negative electrostatic repulsion. An exception is a small local energy minimum found for a pyridine configuration with *γ* = 0° (Table 1). Constructing an optimal pyridine dimer configuration, charge separation in a molecule of pyridine needs to be taken into account as well. This charge separation is relatively high and complex. The HLY charge-fitting analysis shows that in a pyridine molecule the nitrogen atom and some carbon atoms are negatively charged and some carbon atoms are positively charged (Fig. 2a). The calculated dipole moment of a pyridine molecule, using M062X/aug-cc-pVTZ, is also relatively high (Fig. 2b).

**Table 1:** The energy minima for the respective *γ* values (in a function of *β*) found on the basis of PES maps created on the basis of energy minima found for the systems with the given *β* and *d*

*γ* (°)	*α* (°)	*β* (°)	*d* (Å)	Energy (kcal mol^-1^)
0	180	0	3.7	−2.63
	270	20	3.7	−2.78
	0	90	6.3	−2.80
45	120	20	3.7	−3.16
	210	90	6.1	−2.79
90	150	20	3.7	−3.62
	270	90	6.1	−2.22
135	180	20	3.7	−3.68
	330	90	6.2	−2.54
180	120	20	3.7	−3.59
	0	90	6.3	−2.80

**Fig. 6:**
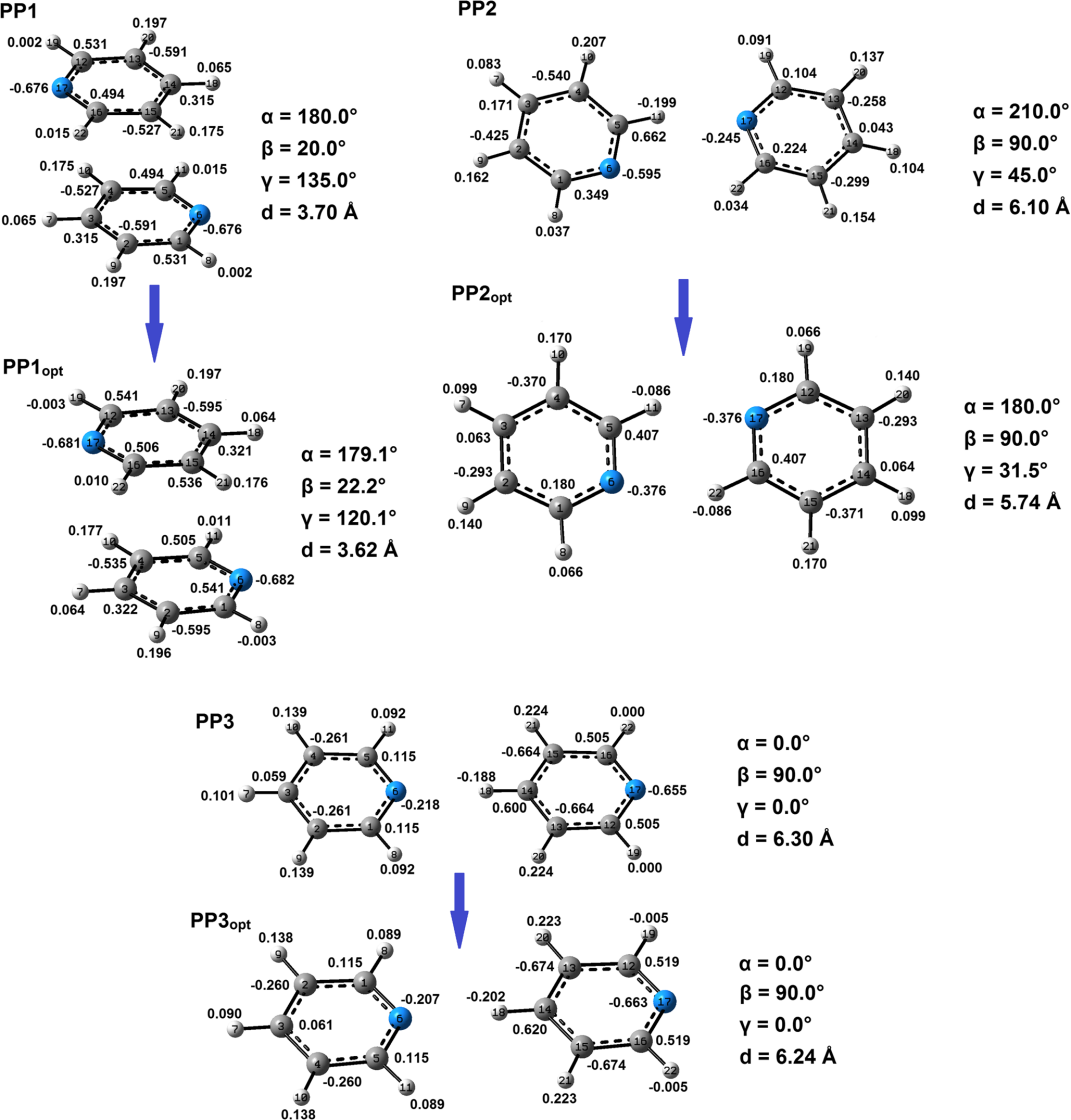
Geometries of the pyridine-pyridine systems that are associated with the found energy minima, together with the atom HLY charge values (e) and the atom numbering scheme (integers) used in NBO analysis. PP1_opt_, PP2_opt_, and PP3_opt_ are the configurations that resulted from the optimization of PP1, PP2, and PP3 systems

Investigating the system interaction energy as a function of *d* and *β* parameters, two main areas associated with energy minima are observed (Fig. 4). The first area corresponds to stacked pyridine dimers and the most stable such configuration was found for *d* = 3.7 Å. In this parallel-displaced configuration (PP1, Fig. 6) one of the rings is twisted around the other about 180°. The second area of energy minima is observed for much larger *d* and *β* (equal to around 6 Å and 90°, respectively, Fig. 4) and it is associated with two pyridine dimer configurations. In both geometries the pyridine molecules can create hydrogen bonds. In the first case one of the pyridine rings is twisted around the other so two hydrogen bonds could be created (PP2, Fig. 6). In the second one no such twist is observed and only one hydrogen bond is formed (PP3, Fig. 6).

B3LYP-D3BJ interaction energy associated with PP1 is lower (more binding) than those calculated for PP2 and PP3. For PP1 the interaction energy is equal to −3.68 kcal mol^-1^. For PP2 and PP3 the interaction energies are similar and they are equal to −2.79 and −2.80 kcal mol^-1^, respectively for PP2 and PP3. In PP1 configuration both pyridine molecules are much further from co-planarity than in PP2 and PP3 where both pyridine monomers are co-planar. Being further from co-planarity (a small *β* angle) increases the pyridine interaction surfaces. Hence, the interaction energy in PP1 is dominated by dispersion energy and this energy is the main cause of the binding interaction in this system (Table 2). In PP1 the contribution of electron correlation energy is around three times greater than it is in PP2 and PP3 (Table 2). Unlike in PP1, the interaction energy in PP2 and PP3 is dominated by electrostatic forces with the E^ele^ about twice the value of E^corr^. This E^ele^ term is even slightly higher than it is in PP1. The same is seen for E^pol^ terms (Table 2).

**Table 2:** The LMO-EDA decomposed energy terms (kcal mol^-1^). *E^disp^ energy terms were calculated as a difference between the B3LYP-D3BJ/DZ energy and HF/DZ energy

			PP1							PP3	PP2	PP1_opt_	PP2_opt_	PP3_opt_
α (°)	180.0	180.0	180.0	150.0	180.0	180.0	210.0	180.0	210.0	0.0	210.0	179.1	180.0	0.0
β (°)	0.0	10.0	20.0	30.0	40.0	50.0	60.0	70.0	80.0	90.0	90.0	22.2	90.0	90.0
γ (°)	0.0	180.0	135.0	90.0	90.0	90.0	90.0	45.0	45.0	0.0	45.0	120.1	31.5	0.0
d (Å)	3.70	3.70	3.70	3.90	4.40	4.90	5.40	5.80	6.00	6.30	6.10	3.62	5.74	6.24
E^ele^	−1.29	−1.80	−2.83	−3.23	−2.67	−2.61	−2.25	−2.20	−3.27	−3.81	−3.94	−3.99	−6.41	−4.08
E^ex^	−8.17	−9.22	−11.95	−11.82	−8.21	−6.87	−5.19	−4.37	−6.05	−6.27	−6.53	−15.35	−11.31	−7.01
E^rep^	13.05	14.55	18.80	18.72	13.22	11.19	8.53	7.20	10.02	10.43	10.84	24.44	18.86	11.70
E^ex^ + E^rep^	4.88	5.33	6.85	6.90	5.01	4.32	3.34	2.83	3.97	4.16	4.31	9.09	7.55	4.69
E^pol^	−0.30	−0.43	−0.72	−0.73	−0.52	−0.44	−0.39	−0.48	−1.02	−1.22	−1.21	−0/94	−1.97	−1.33
E^disp*^	−5.92	−6.22	−6.97	−6.48	−4.39	−3.74	−2.83	−2.28	−2.04	−1.93	−1.95	−8.03	−3.09	−2.08
HF/DZ	3.29	3.10	3.29	2.94	1.81	1.27	0.71	0.16	−0.31	−0.87	−0.84	4.16	−0.83	−0.72
B3LYP-D3BJ/DZ	−2.63	−3.12	−3.68	−3.54	−2.58	−2.47	−2.24	−2.12	−2.35	−2.80	−2.79	−3.87	−3.92	−2.80

The results are shown for the systems corresponding to energy minima found for the given *β* as well as for PP1, PP2, PP3, PP1_opt_, PP2_opt_, and PP3_opt_ configurations

Although the interaction energies of approximately 25,000 different pyridine dimers have been calculated, the resolution of the created PES maps was restricted to the selected geometrical parameters (Fig. 2c). The optimization of PP1, PP2, and PP3 resulted in system configurations PP1_opt_, PP2_opt_, and PP3_opt_, respectively (Fig. 6). The dependencies between the energy terms in the optimized and non-optimized configurations are similar. However, the interaction energies between monomers in the former are lower (more binding) compared to those calculated for the latter. The exception is PP3_opt_ with the interaction energy between monomers equal to that in PP3. The biggest difference (around 1 kcal mol^-1^) is seen comparing PP2_opt_ to PP2 (Table 2). As it was found, the interaction energies for hydrogen bonded pyridine molecules strongly depend on *α* and *γ.* Even small changes of these parameters can drastically change the interaction energy—especially when a dimer with two C-H···N hydrogen bonds is considered (Fig. 6). This occurs for *α* equal to around 180° and *γ* in the range of 30–40° (Fig. 5). As *α* approaches 180° the second monomer twists so the creation of two identical C-H···N hydrogen bonds becomes possible—a dimer system with one hydrogen bond is converted to a system with two hydrogen bonds. It is only possible with the increase in *γ*. With *α* = 180° and *γ =* 0° both nitrogen atoms face each other which leads to a strong electrostatic repulsion and the binding interaction becomes impossible. With the further increase in *α* and *γ* a system is converted back to a system with only one C-H···N hydrogen bond—the rotation angle (*γ*) needs to be adjusted to prevent a system from repulsive H···H interactions.

**Fig. 7:**
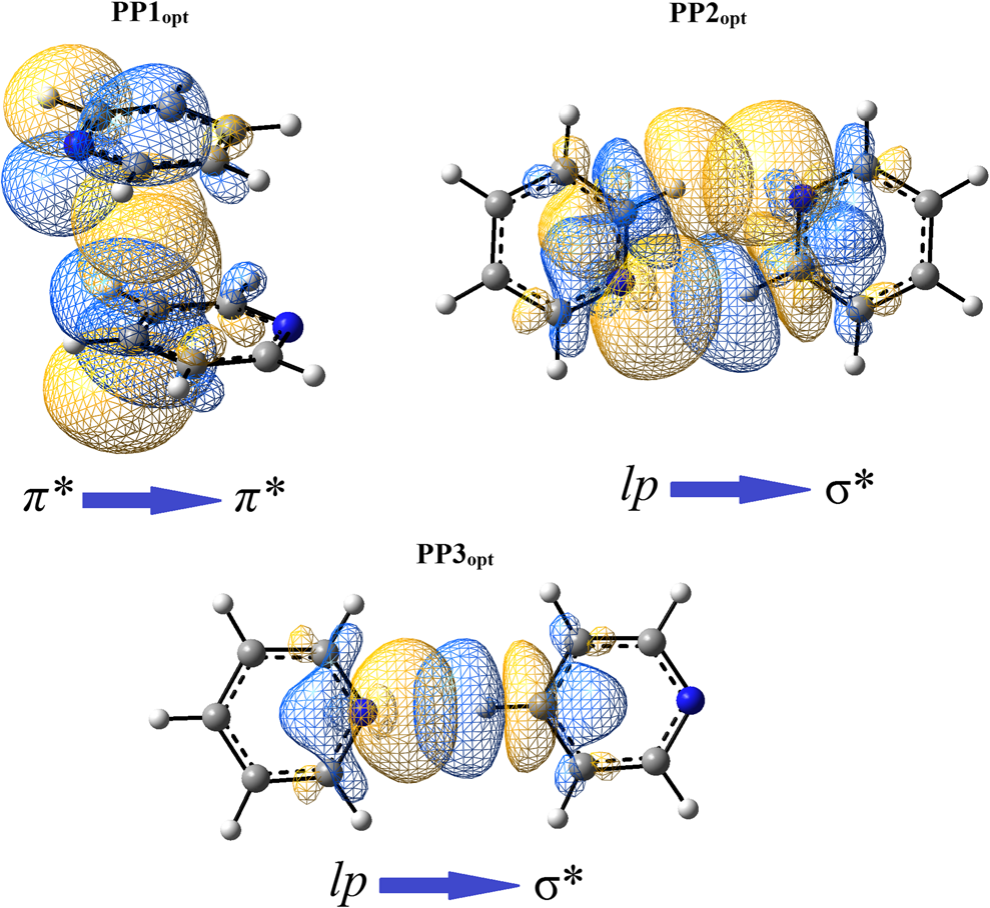
PP1_opt_, PP2_opt_, and PP3_opt_ configurations of pyridine dimers with the selected NBO orbitals (drawn with the isovalue equal to 0.015 au) taking part in the intermolecular interactions

Considering the binding of pyridine molecules in orbital terms, the importance of some interactions associated with the overlap of the molecular orbitals is seen. In PP1 and PP1_opt_ systems, the NBO analysis underlies the importance of π···π interactions. It has been found that the most significant interactions are between antibonding π orbitals present in C-N bonds in one monomer with antibonding π orbitals in C-C bonds in the second monomer (Table 3). These interactions involve the transfer of electron density from antibonding π orbitals of C-N bonds in one monomer to antibonding π orbitals of C-C bonds in the second monomer. This finding may be validated by the performed charge analysis. In PP1 and PP1_opt_ the charge differences between atoms of C-N bonds are slightly higher than they are in a non-interacting pyridine. The same is observed for C-C bonds. In PP1 and PP1_opt_ they are slightly more polarized than they are in a non-interacting pyridine (Figs. 2a and 6). The estimation of energetic importance of those π orbital interactions by second-order perturbation theory (as implemented in NBO) shows that their energy contribution is relatively high. However, it is much higher in PP1_opt_ than it is in PP1 (Table 3). This suggests that the interactions related to π orbitals overlapping (Fig. 7) are highly dependent on geometry. Nevertheless, for those two systems they are a significant source of the binding interactions—direct interactions between the respective molecular π orbitals add an extra energy (Table 3) making the interactions between the pyridine monomers the binding one. Other π orbital interactions (e.g., the ones associated with the transfer of electron density from bonding π orbitals of C-C bonds in one monomer to antibonding π orbitals of C-C bonds in the second monomer) are present as well but they are less significant (Table 3).

**Table 3:** The second order perturbative estimates of donor-acceptor interactions in the NBO basis for the selected pyridine dimer systems

Donor unit		Acceptor unit		Stabilization energy (kcal mol^-1^)
Type	Donor NBO	Type	Acceptor NBO	
PP1				
π	C1–C2	π*	C14–C15	0.32
π	C5–N6	π*	C14–C15	0.22
π*	C5–N6	π*	C12–C13	1.26
π*	C5–N6	π*	C14–C15	1.08
π	C12–C13	π*	C3–C4	0.32
π	C16–N17	π*	C3–C4	0.22
π*	C16–N17	π*	C1–C2	1.26
π*	C16–N17	π*	C3–C4	1.08
PP1_opt_				
π	C3–C4	π*	C16–N17	0.24
π	C1–C2	π*	C14–C15	0.37
π*	C5–N6	π*	C12–C13	1.34
π*	C5–N6	π*	C14–C15	1.90
π	C12–C13	π*	C3–C4	0.36
π	C14–C15	π*	C5–N6	0.23
π*	C16–N17	π*	C1–C2	1.24
π*	C16–N17	π*	C3–C4	2.05
PP2				
lp	N6	ry*	H22	0.39
lp	N17	σ*	C5–H11	2.48
PP2_opt_				
lp	N6	ry*	H22	0.20
lp	N6	σ*	C16–H22	2.08
lp	N17	ry*	H11	0.20
lp	N17	σ*	C5–H11	2.07
PP3				
lp	N6	σ*	C14–H18	2.62
σ	C14–H18	ry*	N6	0.24
PP3_opt_				
lp	N6	σ*	C14-H18	2.97
σ	C14-H18	ry*	N6	0.27

Only the interactions of energy equal to at least 0.2 kcal mol^-1^ were included. The asterix (*) stands for an antibonding orbital; lp – lone electron pair; ry – Rydberg orbital

For PP2, PP3, PP2_opt_, and PP3_opt_ systems, the NBO analysis validates the creation of C-H···N hydrogen bonds (Table 3). The main source of the interactions involves the orbital interactions utilizing nitrogen’s lone electron pairs and antibonding orbitals of the respective C-H bonds: the electron density of lone electron pairs of nitrogen atoms is transferred to the antibonding orbitals of the respective C-H bonds (Table 3, Fig. 7). HLY charge fitting method confirms this finding. Compared to a non-interacting pyridine, in PP2, PP3, PP2_opt_, and PP3_opt_ systems nitrogen atoms are less negative while the respective hydrogen atoms (taking part in formation of hydrogen bonds) are negative instead of being positive as it is in a non-interacting pyridine molecule (Fig. 6).

**Fig. 8:**
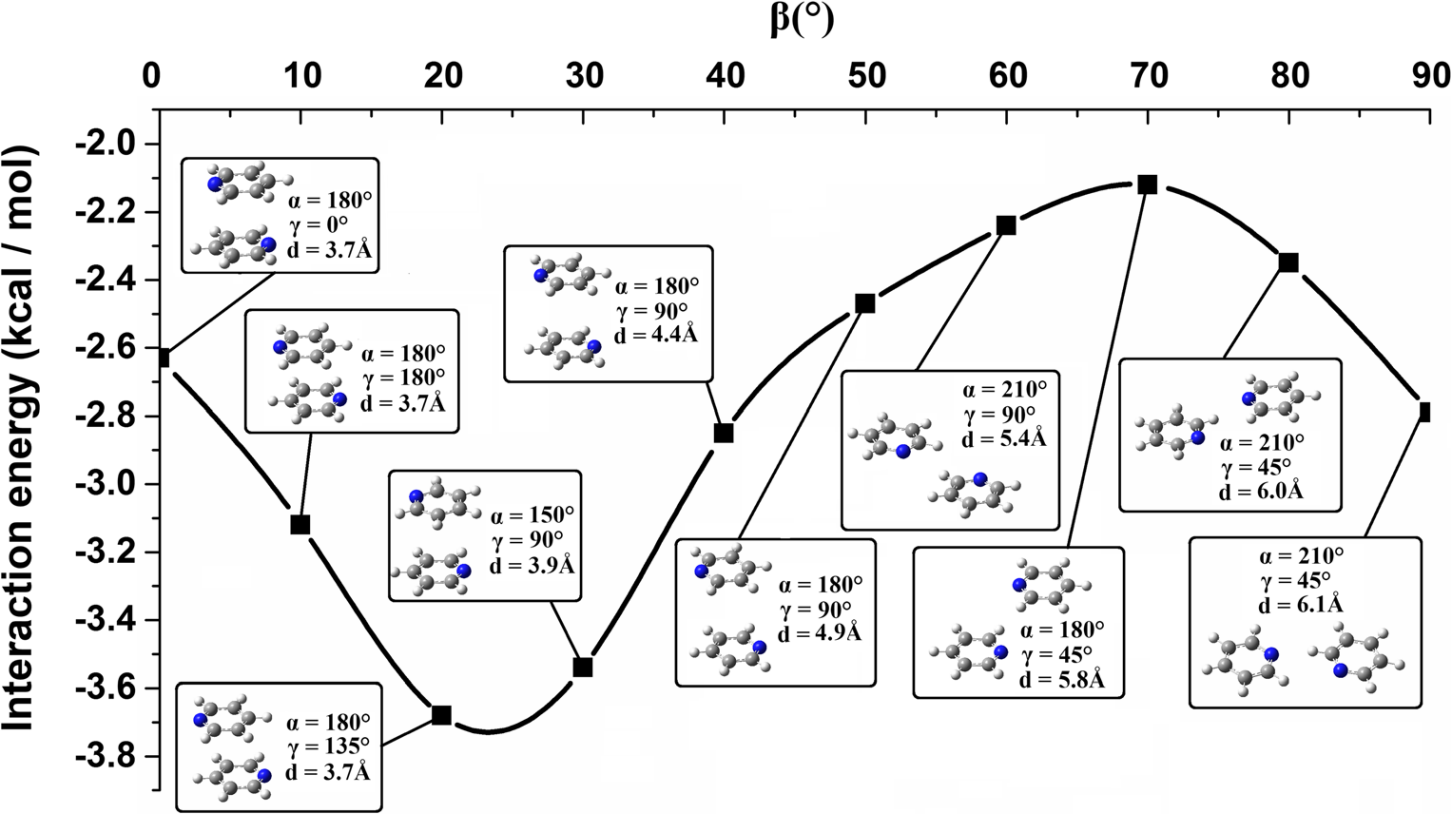
Interaction energy curve created on the basis of data corresponding to energy minima found for the systems with the given *β*. The system configurations are presented in the picture. The curve was created using splicing interpolation

With the increase in *d*, the maximum binding energy for that *d* is observed for a system configuration with higher *β*. It is related to the maximization of dispersion forces. Keeping the same *d*, the increase in *β* causes the decrease in the distance between surfaces of the interacting molecules. Moving from stacked to hydrogen bonded systems via *d* and *β* parameters the changes of *γ* angle are observed (Table 2, Fig. 8). The higher *d* and *β* parameters are, the smaller *γ* becomes (Table 2). Similarly, with the increase in *d* and *β* angles, the contribution of electron correlation energy gets smaller (Table 2). This is related to the decrease of the monomer interaction surfaces. Hence, going to hydrogen bonded systems involves the increase of the interaction energy between the monomers and the interaction energy gets less binding. Though the increase of the interaction energy is observed it does not get higher than −2 kcal mol^-1^ (Fig. 8). This increase is observed till *β* gets close to 70°. After this point (with *β* equal to around 70°) the interaction energy between the monomers starts getting smaller (Table 2, Fig. 8) which is explained by the creation of C-H···N hydrogen bond/bonds.

**Fig. 9:**
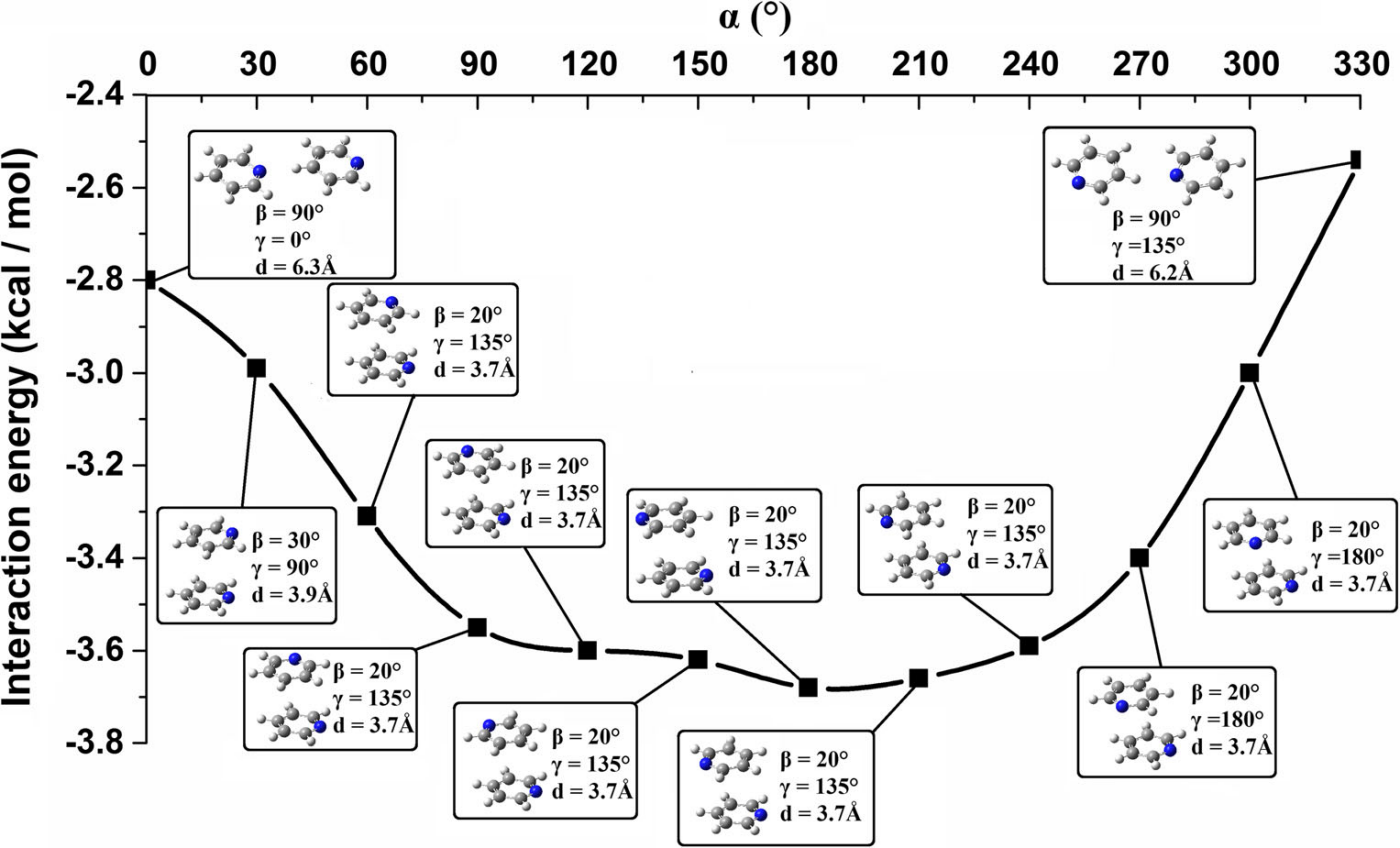
Interaction energy curve created on the basis of data corresponding to energy minima found for the systems with the given *α*. The system configurations are presented in the picture. The curve was created using splicing interpolation

Analyzing the system interaction energy as a function of *α* and *β* parameters (the systems that correspond to energy minima found for the given *α* and *β* values) one main energy minimum is found (Fig. 4). This minimum is quite broad and this enables a certain degree of flexibility in constructing the system configuration of minimum binding energy. This area is within a *β* range of 20–30° and within an *α* range of 60–270°. The interaction energies between pyridine monomers in it are very small and they are close to −3.6 kcal mol^-1^ (Figs. 4 and 9). The distances between the monomer centroids are similar to each other and are within the range 3.7–3.9 Å (Table 4). The *γ* parameters correlate with *α*: going through systems associated with energy minima found for the given *α*, the energy minimization for a system with higher *α* is achieved by the increase of *γ* (Table 4, Fig. 9). The decrease of the system energy is achieved by a molecular arrangement in which there is an optimal balance between the electrostatic and dispersion forces. When a pyridine dimer geometry is kept within some geometrical boundaries, like for example *d* and *β* (Figs. 4 and 9), the maintenance of the system energy may be achieved by changing other parameters. It is possible to result in a parallel-displaced system configuration possessing similar energy but with different twists and rotation angles. In all the systems corresponding to energy minima found for the given *α*, the contribution of electron correlation energy is dominant, and it is around 2.5 times the value of electrostatic energy (Table 4). E^pol^ terms in those systems are similar and they are equal to around −0.7 kcal mol^-1^. In their case, as it was in PP1 and PP1_opt_, the importance of π···π orbital interactions is shown. Again, as found for PP1 and PP1_opt_, these interactions involve the transfer of electron density from antibonding π orbitals of C-N bonds in one monomer to antibonding π orbitals of C-C bonds in the second monomer. Other π orbital interactions (e.g., the ones associated with the transfer of electron density from bonding π orbitals of C-C bonds in one monomer to antibonding π orbitals of C-C bonds in the second monomer) are less important. The most significant π orbital interactions (possessing the highest stabilization energies) are observed for configurations with the twist angle in the range 120–300° (Table 1 in the Supporting information). A special role of the π orbitals in molecular stacking was brought up by Grimme in his work “Do special noncovalent π–π stacking interactions really exist?” [71]. It has been concluded that caution is required describing the effect of π-systems in chemical or biological systems such as stacked nucleobases since the interaction energies of small systems possessing one or two rings, for both aromatic and saturated compounds, are very similar [71]. In another study [72], the orbital interactions in stacked systems were examined in terms of qualitative analysis of the overlap of the monomer densities providing a more intuitive picture of the interaction [72]. In the present study however, NBO analysis has enabled creation a “quantitative picture” of pyridine orbital interactions showing their important role in intermolecular bonding.

**Table 4:** The LMO-EDA decomposed energy terms (kcal mol^-1^). *E^disp^ energy terms were calculated as a difference between the B3LYP-D3BJ/DZ energy and HF/DZ energy

α (°)	0	30	60	90	120	150	180	210	240	270	300	330
β (°)	90	30	20	20	20	20	20	20	20	20	20	90
γ (°)	0	90	135	135	135	135	135	135	180	180	180	135
d (Å)	6.3	3.9	3.7	3.7	3.7	3.7	3.7	3.7	3.7	3.7	3.7	6.2
E^ele^	−3.81	−2.70	−2.39	−2.64	−2.73	−2.80	−2.83	−2.72	−2.68	−2.44	−2.05	−2.70
E^ex^	−6.27	−11.96	−11.66	−11.95	−12.19	−12.25	−11.95	−11.64	−12.04	−11.82	−11.64	−5.28
E^rep^	10.43	18.97	18.34	18.77	19.17	19.27	18.80	18.29	18.92	18.60	18.34	8.67
E^ex^ + E^rep^	4.16	7.01	6.68	6.82	6.98	7.02	6.85	6.65	6.88	6.78	6.70	3.39
E^pol^	−1.22	−0.71	−0.69	−0.75	−0.77	−0.75	−0.72	−0.70	−0.75	−0.72	−0.67	−0.95
E^disp*^	−1.93	−6.58	−6.91	−6.98	−7.08	−7.08	−6.97	−6.89	−7.04	−7.02	−6.99	−2.29
HF/DZ	−0.87	3.59	3.60	3.43	3.48	3.46	3.29	3.23	3.45	3.62	3.99	−0.25
B3LYP-D3BJ/DZ	−2.80	−2.99	−3.31	−3.55	−3.60	−3.62	−3.68	−3.66	−3.59	−3.40	−3.00	−2.54

The results are shown for the systems corresponding to energy minima found for the given *α*

The changes of the interaction energy in a function of *d* and *β* parameters observed for different *γ* parameters (corresponding to energy minima found for the given *α* and *β* and for the given *γ*) are similar to each other (Fig. 3). They are also similar to the changes observed on the basis of the analysis covering all the calculated systems (Fig. 4). In the case of each *γ* angle, two areas of energy minima are observed (Fig. 3). The first one is seen for smaller *d* (in the range of 3.5–4.0 Å) and for smaller *β* (in the range of 0–30°). The second is seen for bigger *d* (in the range of 5.5–7.0 Å) and for bigger *β* (in the range of 70–90°). The first area includes the energy minimum associated with a stacked configuration and for each rotation angle it was found for the same *β* and *d* (Table 1). The interaction energy for this minimum gets more binding as *γ* approaches 135°. The *α* for this minimum changes as well and it gets bigger as *γ* approaches 135° (Table 1). An exception is found for *γ* = 0° for which this energy minimum exists for *α* = 270°. The reason for these different twist angle values is the maximization of the electrostatic binding forces. The second area of energy minima is associated with the formation of C-H···N hydrogen bonds (Table 3, Fig. 6).

Comparing PES maps depicting the interaction energy as a function of *α* and *β* parameters that were made separately for each rotation angle (each PES map corresponds to the interaction energies calculated for the given *γ*) some differences are seen. Different *γ* angles force different geometrical arrangements of the interacting monomers (e.g., two systems with the same *α* but with different *γ* may have configurations with a different distance between nitrogen atoms). However, apart from some differences found for the obtained PES maps, when a stacked geometry is considered, the optimal *β* and *d* for each *γ* appeared to be 20° and 3.7 Å, respectively for *β* and *d* (Table 5). The same was observed for the analysis based on *d* and *β* parameters (Fig. 3, Table 1). For those *β* and *d*, the optimal balance between the contribution of electrostatic and dispersion forces may be achieved. The observed energy minima that corresponds to the parallel-displaced configuration are quite broad (Fig. 3) and this enables, as noticed earlier, a certain degree of flexibility in constructing the system configuration of minimum binding energy. Hence, the twist angle may differ. This is also true for other system configurations. Especially for those associated with *β =* 60° (Fig. 4), which may be considered as systems with C-H···π interactions. In their case, the twist angle of one monomer around the other does not change the energy significantly unless both monomers are in a configuration where both nitrogen atoms are relatively close to each other which leads to strong electrostatic repulsion (E^ele^ term is positive in the *α* range of 150–180°) — which cannot be overcome by binding forces (Tables 6 and 7). The same is observed for higher *β* angles (up to 90°) and geometries for which two nitrogen atoms face each other, except for the configurations for which C-H···N hydrogen bonds can be created (the observed energy minima for *β =* 90°). It needs to be pointed out that for some *γ* some configurations are symmetrically equivalent. For *γ* = 0° the direction of the first monomer lateral movement (associated with the increase in *d* and *β*, Fig. 2) was in accordance with the direction of the vector created between the second monomer centroid and the nitrogen atom of this monomer (the vector beginning was the position of the second monomer centroid). Hence, some pyridine dimers are symmetrically equivalent. A similar situation is found for systems with *γ* = 180° for which the direction of the one monomer lateral movement was opposite to the direction of the vector created between the second monomer centroid and the nitrogen atom of this monomer—the vector beginning was the position of the second monomer centroid.

**Table 5:** The energy minima for the respective *γ* values (in a function of *α*) found on the basis of PES maps created on the basis of energy minima found for the systems with the given *α* and *β*

*γ* (°)	*α* (°)	*β* (°)	*d* (Å)	Energy (kcal mol^-1^)
0	0	90	6.3	−2.80
	90	20	3.7	−2.78
	180	0	3.7	−2.63
45	270	20	3.7	−2.78
	120	20	3.7	−3.16
90	150	20	3.7	−3.62
135	180	20	3.7	−3.68
180	0	90	6.3	−2.80
	120	20	3.7	−3.59
	150	20	3.7	−3.59
	180	20	3.7	−3.59
	210	20	3.7	−3.59
	540	20	3.7	−3.59

**Table 6:** The second order perturbative estimates of donor-acceptor interactions in the NBO basis for systems corresponding to energy minima found for the systems with the given *α* values and with *β* = 60° and for *γ* = 0°

Donor unit		Acceptor unit		Stabilization energy (kcal mol^-1^)
Type	Donor NBO	Type	Acceptor NBO	
α = 0°, d = 5.6 Å				
lp	N6	π*	C14–C15	0.20
π*	C1–N6	π*	C14–C15	0.28
α = 30°, d = 5.5 Å				
π*	C5–N6	π*	C13–C14	0.30
α = 60°, d = 5.6 Å				
π*	C5–N6	π*	C14–C15	0.25
α = 90°, d = 5.5 Å				
π*	C5–N6	π*	C14–C15	0.32
α = 120°, d = 5.5 Å				
lp	N6	π*	C16–N17	0.25
π*	C5–N6	π*	C16–N17	1.38
α = 150°, d = 5.4 Å				
π*	C5–N6	π*	C15–C16	0.58
α = 180°, d = 6.4 Å				
no interaction of energy equals to at least 0.2 kcal mol^-1^				
α = 210°, d = 5.4 Å				
π*	C1–N6	π*	C12–C13	0.59
α = 240°, d = 5.5 Å				
lp	N6	π*	C12–N17	0.26
π*	C1–N6	π*	C12–N17	1.38
α = 270°, d = 5.5 Å				
π*	C1–N6	π*	C13–C14	0.32
α = 300°, d = 5.6 Å				
π*	C1–N6	π*	C13–C14	0.25
α = 330°, d = 5.5 Å				
π*	C1–N6	π*	C14–C15	0.30

Only the interactions of energy equal to at least 0.2 kcal mol^-1^ were included. The asterix (*) stands for an antibonding orbital; lp – lone electron pair

**Table 7:** The LMO-EDA decomposed energy terms (kcal mol^-1^). *E^disp^ energy terms were calculated as a difference between the B3LYP-D3BJ/DZ energy and HF/DZ energy

α (°)	0	30	60	90	120	150	180	210	240	270	300	330
d (Å)	5.6	5.5	5.6	5.5	5.5	5.4	6.4	5.4	5.5	5.5	5.6	5.5
E^ele^	−0.32	−0.16	−0.14	−0.21	−0.38	0.63	1.11	0.63	−0.39	−0.21	−0.14	−0.16
E^ex^	−2.78	−3.17	−2.89	−3.01	−3.14	−3.05	−0.09	−3.05	−3.14	−3.01	−2.89	−3.17
E^rep^	4.47	5.04	4.63	4.78	5.06	4.83	0.14	4.83	5.06	4.78	4.63	5.04
E^ex^ + E^rep^	1.69	1.87	1.74	1.77	1.92	1.78	0.05	1.78	1.92	1.77	1.74	1.87
E^pol^	−0.30	−0.29	−0.29	−0.28	−0.34	−0.35	−0.07	−0.35	−0.34	−0.28	−0.29	−0.29
E^disp*^	−2.17	−2.55	−2.29	−2.41	−2.23	−2.50	−0.82	−2.50	−2.23	−2.41	−2.29	−2.55
HF/DZ	1.07	1.42	1.31	1.28	1.19	2.06	1.10	2.06	1.19	1.28	1.31	1.42
B3LYP-D3BJ/DZ	−1.1	−1.13	−0.98	−1.13	−1.04	−0.44	0.28	−0.44	−1.04	−1.13	−0.98	−1.13

The results are shown for systems corresponding to energy minima found for the selected systems with *β* = 60° (on the basis of PES created in a function of *α* and *β* parameters for *γ* = 0°)

**Fig. 10:**
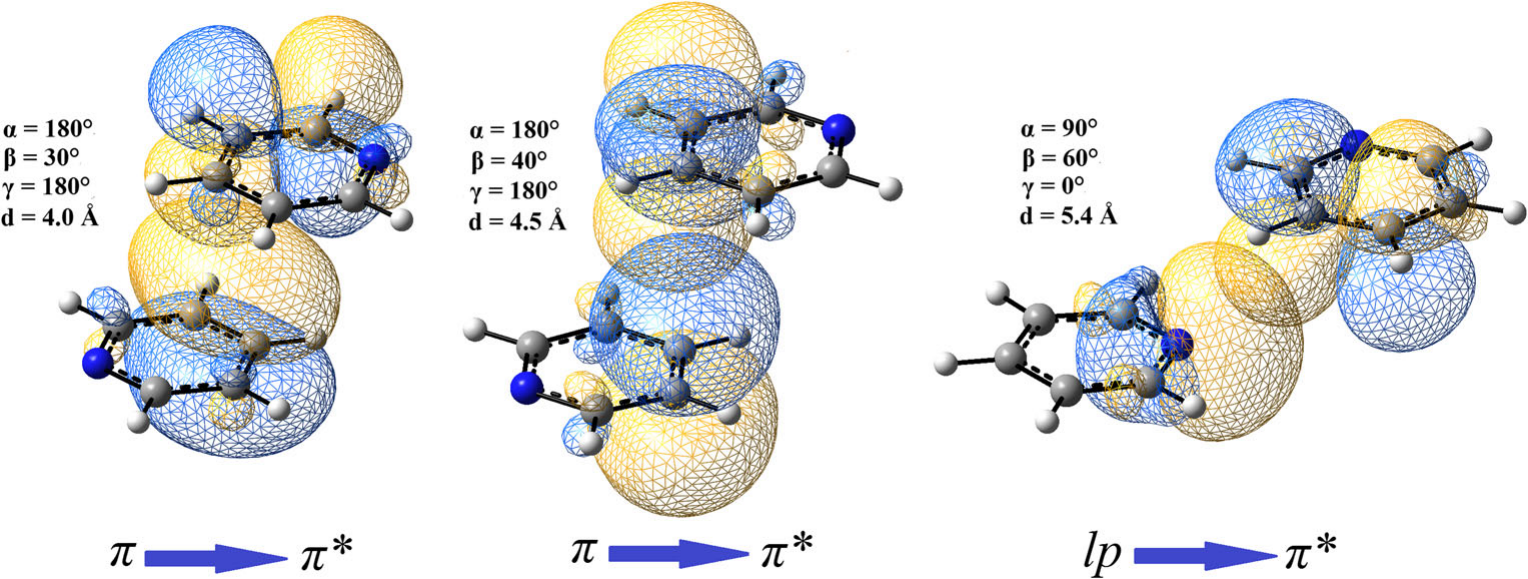
Selected configurations of pyridine dimers where the formation of C-H···π interactions might be proposed. The selected NBO orbitals (drawn with the isovalue equal to 0.015 au) taking part in the intermolecular interactions are shown

Some pyridine dimer systems (for example those with *β =* 60°) may be considered as systems with C-H···π interactions (Fig. 10). However, NBO analysis does not validate this claim. This also appeared to be true for dimers with lower *β* where a C-H bond of one monomer is closer to the centroid of the second one. The most significant interactions are those involving antibonding π orbitals and nitrogen’s lone electron pairs (Table 6). Even considering orbital interactions of relatively small energies (down to 0.05 kcal mol^-1^), the interactions that could be assigned as C-H···π appeared to be considered as insignificant (Table 2 in the Supporting information).

## Conclusions

The appliance of multidimensional analysis can provide much deeper insight into the “landscape” of the interaction energies of the particular systems and enables one to see “a wider picture” of what really occurs. The combination of many different methods has revealed the complexity of the stacking interactions. Apart from providing a “literal new look” into pyridine stacking patterns another picture has emerged. A stacking interaction in pyridine dimer systems “is seen” as a combination of many different sources of energy, including orbital ones, which is true for many different geometries.

The binding geometrical boundaries of the stacked pyridine systems are relatively broad (Figs. 3 and 4) and this provides a great degree of flexibility in the system configuration. It is possible to result in a system configuration possessing similar energy but with different geometrical parameters. Hence, when a system is constrained to some geometrical parameters (e.g., the distance between the ring centroid and the lateral shifting of one ring along the plane of the other) it is possible to maintain the system interaction energy changing other parameters. It may give one great adjustability in the crystal engineering processes.

The “picture” of the interplay of electrostatic forces and electron correlation is clearly seen. The electron correlation effects are important for the majority of pyridine dimer geometries, even for those where the formation of C-H···π interactions might be proposed. The electrostatic energy is far more significant in planar dimer configurations (*β ≈ 9*0°) for which the formation of C-H···N hydrogen bonds is plausible.

The important role of π orbitals in pyridine stacking has been confirmed. For this system, π-orbital interactions provide additional binding force to combine the two monomers when they are close enough to each other. This is true even for the geometries where the formation of C-H···π interactions might be proposed instead.

The calculated B3LYP-D3BJ interaction energies, corresponding to the found pyridine dimer configurations associated with the energy minima (PP1_opt_ and PP2_opt_, Table 2), are very close to those reported earlier, both for stacked and hydrogen bonded pyridine systems, being respectively equal to −3.90 and −4.15 kcal mol^-1^ [52].

It is expected that the presented results will shed more light on stacking interaction phenomenon leading to a deeper understating of its role in natural (e.g., biological) and engineered systems. They may be of key importance in crystal structure prediction, the habit of a crystal itself, and hence, they may even find a use in modeling of a crystal possessing desired properties. The obtained PES maps (together with the performed analyses) may serve as a guide for qualitative predictions of the existence of binding interactions; even for relatively long distances between the monomer centroids (up to 7 Å). The presented study may also inspire other researchers to engage in an even deeper study of NCI phenomenon helping to understand its physics and role in a solid state as well as leading us closer to understanding the hierarchy of intermolecular interactions in a molecular crystal and biological systems.

## Electronic supplementary material

ESM 1 (DOCX 18.2 kb)
