# Supplementary material for: Discovering the stacking landscape of a pyridine-pyridine system
Source: J Mol Model. 2017 Nov 9;23(12):338. doi: 10.1007/s00894-017-3496-4 (PMC5680376; doi:10.1007/s00894-017-3496-4)
Supplement: Supplementary file 1 — (DOCX 18.2 kb) [file 894_2017_3496_MOESM1_ESM.docx]

Discovering the stacking landscape of a pyridine-pyridine system

Tomasz Sierański

Institute of General and Ecological Chemistry

*Lodz University of Technology,* *Zeromskiego 116, 90-924, Lodz, Poland*

*Tomasz Sierański*

*Institute of General and Ecological Chemistry*

*Lodz University of Technology*

*Zeromskiego 116*

*90-924 Lodz, Poland*

Phone number: *+ 48 42 631 31 11*

Fax number*:+48 42 631-31-03*

Email address: [*tomasz.sieranski@p.lodz.pl*](mailto:tomasz.sieranski@p.lodz.pl)

Table 1. The second order perturbative estimates of donor-acceptor interactions in the NBO basis for systems corresponding to energy minima found for the systems with the given α. Only the interactions of energy equal to at least 0.2 kcal/mol were included. The asterix (*) stands for an antibonding orbital; lp – lone electron pair; ry – Rydberg orbital.

|  | | **Acceptor unit** | | **Stabilization energy**  **(kcal/mol)** |
| --- | --- | --- | --- | --- |
| **Type** | **Donor NBO** | **Type** | **Acceptor NBO** |  |
| **α = 0° (β = 90°, γ = 0°, d = 6.3 Å)** | | | | |
| lp | N6 | σ* | C14–H18 | 2.62 |
| σ | C14–H18 | ry* | N6 | 0.24 |
| **α = 30° (β = 30°, γ = 90°, d = 3.9 Å)** | | | | |
| π | C5–N6 | π* | C12–N17 | 0.37 |
| π* | C5–N6 | π* | C13–C14 | 0.47 |
| π | C12–N17 | π* | C5–N6 | 0.27 |
| **α = 60° (β = 20°, γ = 135° , d = 3.7 Å)** | | | | |
| π | C5–N6 | π* | C12–N17 | 0.32 |
| π | C12–N17 | π* | C5–N6 | 0.27 |
| **α = 90° (β = 20°, γ = 135°, d = 3.7 Å)** | | | | |
| π | C5–N6 | π* | C13–C14 | 0.22 |
| π | C12–N17 | π* | C3–C4 | 0.22 |
| **α = 120° (β = 20°, γ = 135° , d = 3.7 Å)** | | | | |
| π | C1–C2 | π* | C13–C14 | 0.37 |
| π* | C5–N6 | π* | C13–C14 | 1.22 |
| π | C12–N17 | π* | C3–C4 | 0.24 |
| π | C15–C16 | π* | C3–C4 | 0.30 |
| π* | C12–N17 | π* | C3–C4 | 0.36 |
| **α = 150° (β = 20°, γ = 135°, d = 3.7 Å)** | | | | |
| π | C5–N6 | π* | C14–C15 | 0.22 |
| π* | C5–N6 | π* | C12–C13 | 2.14 |
| π | C12–C13 | π* | C5–N6 | 0.28 |
| π | C12–C13 | π* | C3–C4 | 0.31 |
| π* | C16–N17 | π* | C3–C4 | 1.71 |
| **PP1** | | | | |
| π | C1–C2 | π* | C14–C15 | 0.32 |
| π | C5–N6 | π* | C14–C15 | 0.22 |
| π* | C5–N6 | π* | C12–C13 | 1.26 |
| π* | C5–N6 | π* | C14–C15 | 1.08 |
| π | C12–C13 | π* | C3–C4 | 0.32 |
| π | C16–N17 | π* | C3–C4 | 0.22 |
| π* | C16–N17 | π* | C1–C2 | 1.26 |
| π* | C16–N17 | π* | C3–C4 | 1.08 |
| **α = 210° (β = 20°, γ = 135°, d = 3.7 Å)** | | | | |
| π | C1–C2 | π* | C16–N17 | 0.39 |
| π* | C5–N6 | π* | C14–C15 | 1.98 |
| π | C16–N17 | π* | C3–C4 | 0.27 |
| π | C14–C15 | π* | C5–N6 | 0.32 |
| π* | C16–N17 | π* | C1–C2 | 1.19 |
| **α = 240° (β = 20°, γ = 180°, d = 3.7 Å)** | | | | |
| π | C1–N6 | π* | C14–C15 | 0.22 |
| π | C12–C13 | π* | C2–C3 | 0.32 |
| π* | C16–N17 | π* | C2–C3 | 1.35 |
| **α = 270° (β = 20°, γ = 180°, d = 3.7 Å)** | | | | |
| π | C4–C5 | π* | C13–C14 | 0.20 |
| π | C4–C5 | π* | C15–C16 | 0.34 |
| π* | C1–N6 | π* | C15–C16 | 1.10 |
| π | C13–C14 | π* | C4–C5 | 0.27 |
| π* | C12–N17 | π* | C2–C3 | 1.59 |
| **α = 300° (β = 20°, γ = 180°, d = 3.7 Å)** | | | | |
| π | C4–C5 | π* | C14–C15 | 0.31 |
| π | C14–C15 | π* | C4–C5 | 0.29 |
| π* | C16–N17 | π* | C2–C3 | 0.73 |
| π* | C16–N17 | π* | C4–C5 | 0,28 |
| **α = 330° (β = 90°, γ = 135°, d = 6.2 Å)** | | | | |
| lp | N17 | *σ | C4–H10 | 1.82 |

Table 2. The second order perturbative estimates of donor-acceptor interactions in the NBO basis for the selected configurations of pyridine dimers in case of which the formation of C-H···π interactions might be proposed. The interactions of energy equal to at least 0.05 kcal/mol were included. The asterix (*) stands for an antibonding orbital; lp – lone electron pair; ry – Rydberg orbital.

| **Donor unit** | | **Acceptor unit** | | **Stabilization energy**  **(kcal/mol)** |
| --- | --- | --- | --- | --- |
| **Type** | **Donor NBO** | **Type** | **Acceptor NBO** |  |
| **α = 180°, β = 30°, γ = 180°, d = 4.0 Å** | | | | |
| π | C2–C3 | π* | C13–C14 | 0.11 |
| π | C2–C3 | π* | C15–C16 | 0.15 |
| π | C1–N6 | π* | C13–C14 | 0.07 |
| lp | N6 | ry* | H18 | 0.06 |
| π* | C1–N6 | π* | C13–C14 | 0.15 |
| π* | C1–N6 | π* | C15–C16 | 0.11 |
| π | C13–C14 | π* | C2–C3 | 0.11 |
| π | C13–C14 | π* | C4–C5 | 0.15 |
| π | C12–N17 | ry* | C2–C3 | 0.07 |
| lp | N17 | π* | H7 | 0.06 |
| π* | C12–N17 | π* | C2–C3 | 0.15 |
| π* | C12–N17 | π* | C4–C5 | 0.11 |
| **α = 180°, β = 40°, γ = 180°, d = 4.5 Å** | | | | |
| π | C2–C3 | π* | C13–C14 | 0.31 |
| π | C2–C3 | π* | C15–C16 | 0.15 |
| σ | C3–C4 | ry* | C14 | 0.06 |
| π | C13–C14 | π* | C2–C3 | 0.31 |
| π | C13–C14 | π* | C4–C5 | 0.15 |
| σ | C14–C15 | ry* | C3 | 0.06 |
| **α = 90°, β = 60°, γ = 0°, d = 5.4 Å** | | | | |
| π | C5–N6 | π* | C16–N17 | 0.11 |
| σ | C4–H10 | ry* | H22 | 0.06 |
| lp | N6 | π* | C16–N17 | 0.11 |
| lp | N6 | π* | C14–C15 | 0.14 |
| π* | C1–C2 | π* | C14–C15 | 0.20 |
| π* | C5–N6 | π* | C16–N17 | 0.08 |
| π* | C5–N6 | π* | C14–C15 | 0.41 |
| σ | C15–C16 | ry* | N6 | 0.09 |
| σ | C14–H18 | ry* | C1 | 0.05 |
